# Supplementary material for: MOTS‐c promotes phosphorodiamidate morpholino oligomer uptake and efficacy in dystrophic mice
Source: EMBO Mol Med. 2020 Dec 18;13(2):e12993. doi: 10.15252/emmm.202012993 (PMC7863382; doi:10.15252/emmm.202012993)
Supplement: Supplementary file 1 — Appendix [file EMMM-13-e12993-s001.pdf]

MOTS-c promotes phosphorodiamidate morpholino oligomer uptake and efficacy in dystrophic mice

Ning Ran[1 §], Caorui Lin[1 §], Ling Leng[1], Gang Han[2], Mengyuan Geng[1], Yingjie Wu[1], Scott Bittner[3], Hong M Moulton[3], HaiFang Yin[1,4]*

APPENDIX.

Contents: Appendix Figures S1 – S4

Appendix Figure S1 – Page 2

Systemic comparison between PMO-M and PMO in GF (PMO-GF) in adult *mdx* mice under identical conditions.

Appendix Figure S2 – Page 3

Western blot to examine dystrophin expression in the hearts of treated *mdx* mice.

Appendix Figure S3 – Page 4

Comparison between PMO-M and PMO in glycine (PMO-G) in adult *mdx* mice under identical conditions.

Appendix Figure S4 – Page 5

Morphological and pathological assessment of *mdx* mice treated with MOTS-c.

Appendix Table S1 – Page 6

Summary of exact P-values in figures

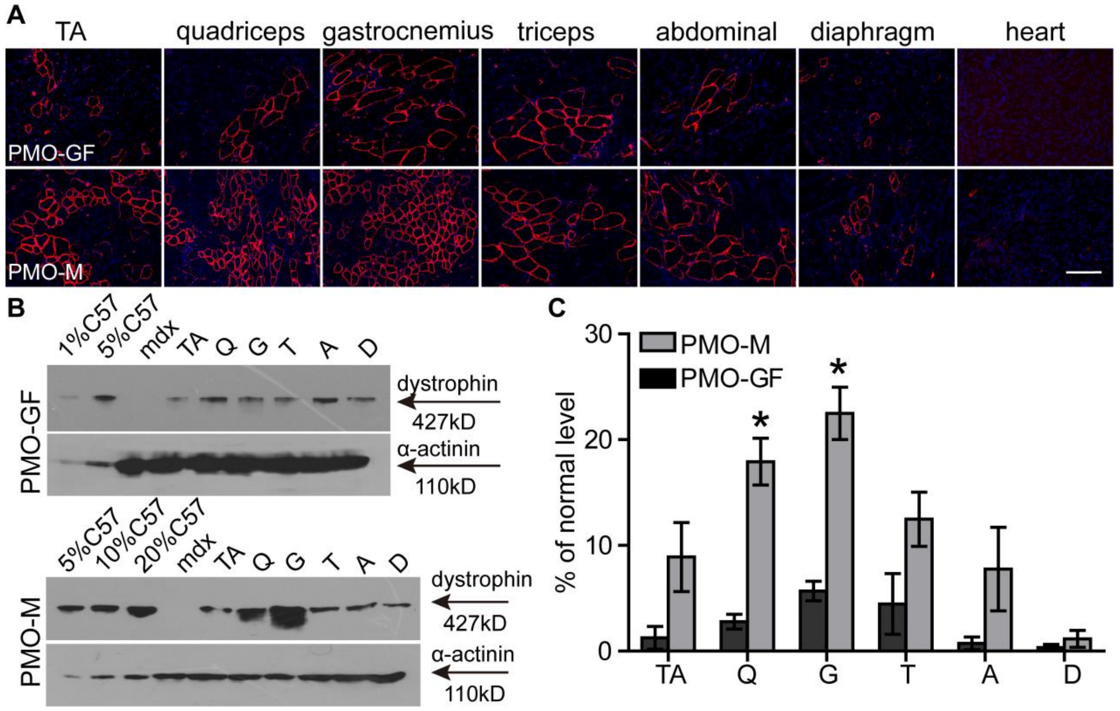

Appendix Figure S1. Systemic comparison between PMO-M and PMO in GF (PMO-GF) in adult *mdx* mice under identical conditions. PMO (12.5 mg/kg/week) mixed with MOTS-c (500 μg) or PMO-GF (12.5 mg/kg/week) was administered into adult *mdx* mice for 3 weeks intravenously, respectively. (**A**) Immunohistochemistry of dystrophin-positive fibers in body-wide muscles from treated *mdx* mice (scale bar=100 μm). Western blot (**B**) and quantitative analysis (**C**) for dystrophin expression in body-wide muscles from treated *mdx* mice (n=3; *p<0.05, two-tailed t test). TA-tibialis anterior, Q-quadriceps, G-gastrocnemius, T-triceps, A-abdominal muscle, D-diaphragm. 0.5 μg, 2.5 μg, 5 μg and 1 μg total protein from *C57BL/6* and 50 μg of muscle samples from untreated and treated *mdx* mice were loaded. α-actinin was used as the loading control. Data were presented as mean±sem.

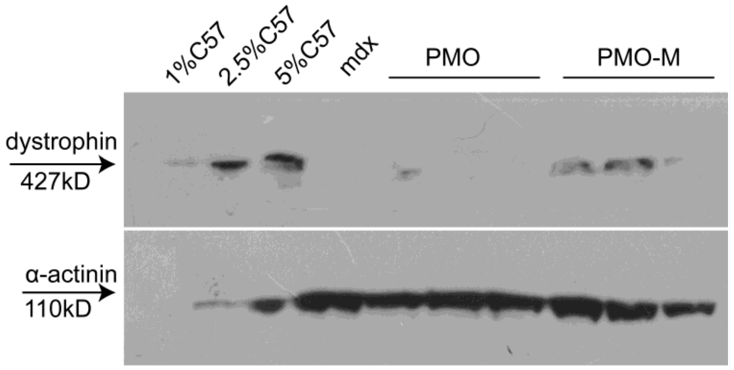

Appendix Figure S2. Western blot to examine dystrophin expression in the hearts of treated *mdx* mice. PMO-M was administered intravenously into adult *mdx* mice at the PMO dose of 12.5 mg/kg/week for 3 weeks followed by 12.5 mg/kg/month for 3 months and tissues were harvested two weeks after last injection. 1 µg, 5 µg and 10 µg total protein from *C57BL/6* and 100 µg of muscle samples from untreated and treated *mdx* mice were loaded. α-actinin was used as the loading control.

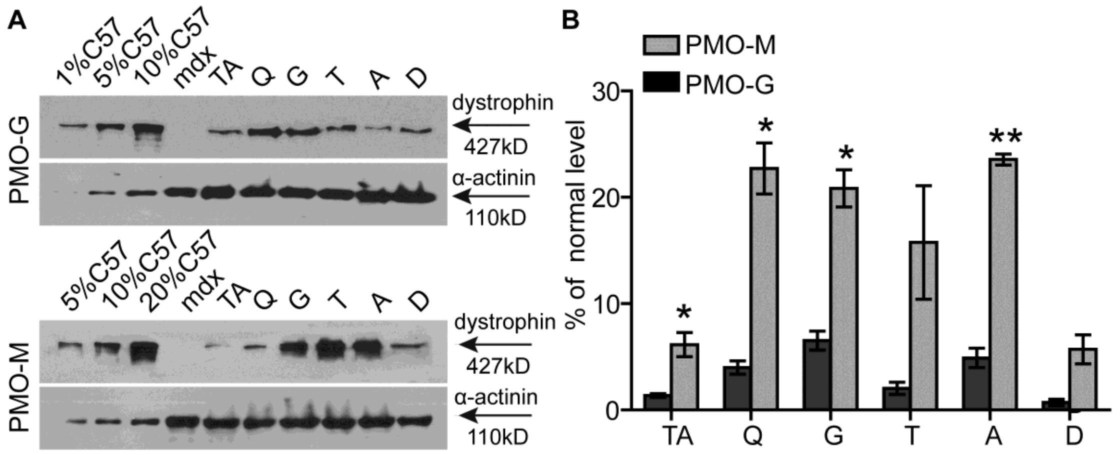

Appendix Figure S3. Systemic comparison between PMO-M and PMO in glycine (PMO-G) in adult *mdx* mice under identical conditions. PMO (25 mg/kg/week) mixed with MOTS-c (500 μg) or PMO-G (25 mg/kg/week) was administered into adult *mdx* mice for 3 weeks intravenously, respectively. Western blot (**A**) and quantitative analysis (**B**) for dystrophin expression in body-wide muscles from *mdx* mice treated with PMO-G (n=4) or PMO-M (n=3) (*p<0.05, **p<0.001, two-tailed t test). The abbreviation is the same as Appendix Figure S1B. 0.5 μg, 2.5 μg, 5 μg and 10 μg total protein from *C57BL/6* and 50 μg of muscle samples from untreated and treated *mdx* mice were loaded. α-actinin was used as the loading control. Data were presented as mean±sem.

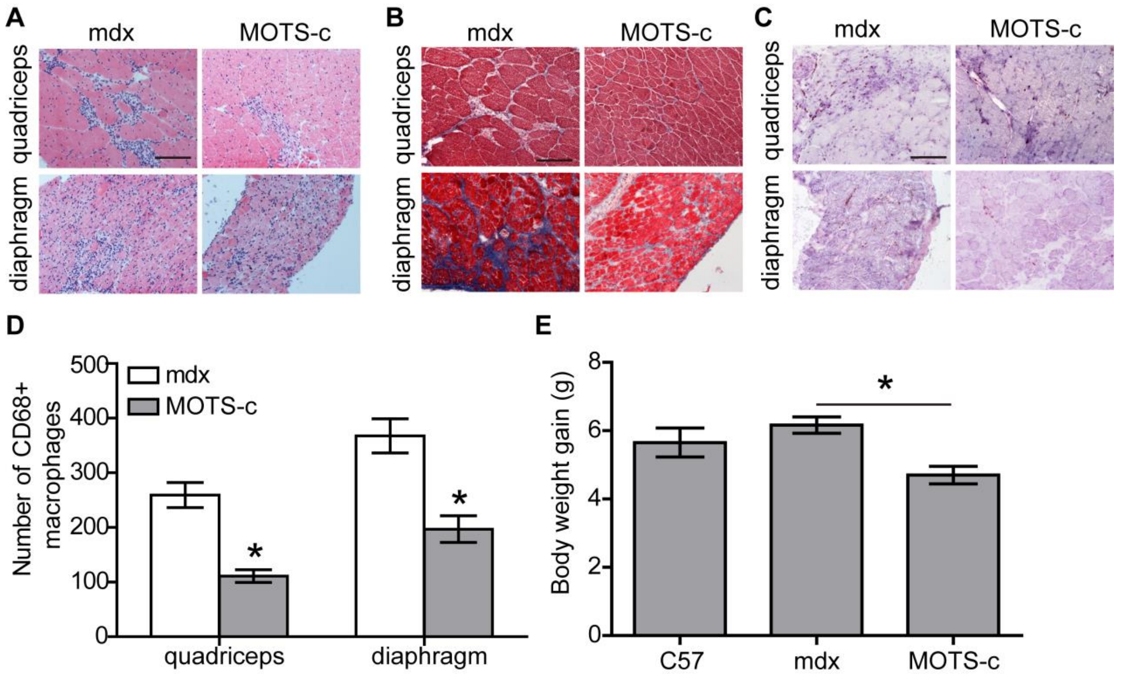

Appendix Figure S4. Morphological and pathological assessment of *mdx* mice treated with MOTS-c. MOTS-c **(**500 μg) was administered into adult *mdx* mice weekly for 3 weeks, followed by monthly injection for 3 months intravenously. Muscles were harvested 2 weeks after last injection. (**A**) H&E staining of quadriceps and diaphragm from treated *mdx* mice (scale bar=100 μm). (**B**) Masson's trichrome staining for quadriceps and diaphragm from treated *mdx* mice (scale bar=100μm). Immunohistochemistry (**C**) and quantitative analysis (**D**) of $CD68^{+}$ macrophages in quadriceps and diaphragm from *mdx* mice treated with MOTS-c (n=4) or untreated *mdx* controls (n=3) (scale bar=100 μm) (*$p<0.05$, two-tailed t test). (**E**) Measurement of body-weight changes of *mdx* mice treated with MOTS-c (n=4), untreated *mdx* (n=3) and wild-type controls (n=3) (*$p<0.05$, One way-ANOVA post hoc Student-Newman-Keuls test). Data were presented as mean ±sem.

Appendix Table S1: Summary of exact p-values in each figure

Figure		comparison	P value	Statistical test
Fig.1A	30min	H2Kmdx v.s H2Kmdx +MOTSc	0.013	two-tailed t test
	40min	H2Kmdx v.s H2Kmdx +MOTSc	0.001	two-tailed t test
	50min	H2Kmdx v.s H2Kmdx +MOTSc	0.011	two-tailed t test
	30min	H2K v.s H2K+MOTSc	0.002	two-tailed t test
	40min	H2K v.s H2K+MOTSc	0.028	two-tailed t test
	50min	H2K v.s H2K+MOTSc	0.006	two-tailed t test
Fig.1B		H2K v.s H2Kmdx	0.005	two-tailed t test
Fig. 1C		NC v.s MOTS-c (H2Kmdx)	0.041	two-tailed t test
Fig. 1D	TA	NC v.s MOTS-c	0.045	two-tailed t test
	Q	NC v.s MOTS-c	0.045	two-tailed t test
	G	NC v.s MOTS-c	0.039	two-tailed t test
	T	NC v.s MOTS-c	0.034	two-tailed t test
	A	NC v.s MOTS-c	0.041	two-tailed t test
	D	NC v.s MOTS-c	0.26	two-tailed t test
	H	NC v.s MOTS-c	0.41	two-tailed t test
	Li	NC v.s MOTS-c	0.00017	two-tailed t test
	S	NC v.s MOTS-c	0.15	two-tailed t test
	Lu	NC v.s MOTS-c	0.02	two-tailed t test
	K	NC v.s MOTS-c	0.14	two-tailed t test
Fig.1E	Q	NC v.s MOTS-c	0.023	two-tailed t test
	D	NC v.s MOTS-c	0.006	two-tailed t test
	A	NC v.s MOTS-c	0.004	two-tailed t test
Fig.2A		Fluorescence intensity	4.448E-07	two-tailed t test
Fig.2C	TA	PMO v.s PMO-M	0.191	two-tailed t test
	Q	PMO v.s PMO-M	0.003	two-tailed t test
	G	PMO v.s PMO-M	0.034	two-tailed t test
	T	PMO v.s PMO-M	0.187	two-tailed t test
	A	PMO v.s PMO-M	0.027	two-tailed t test
	D	PMO v.s PMO-M	0.921	two-tailed t test
	H	PMO v.s PMO-M	0.641	two-tailed t test
	Li	PMO v.s PMO-M	0.932	two-tailed t test
	S	PMO v.s PMO-M	0.463	two-tailed t test
	Lu	PMO v.s PMO-M	0.196	two-tailed t test
	K	PMO v.s PMO-M	0.032	two-tailed t test
Fig.2E		PMO v.s PMO-M	0.000884	two-tailed t test

Fig.2F		PMO v.s PMO-M	0.0143	two-tailed t test
Fig.2I	TA	PMO v.s PMO-M	0.018	two-tailed t test
	Q	PMO v.s PMO-M	0.02	two-tailed t test
	G	PMO v.s PMO-M	0.028	two-tailed t test
	T	PMO v.s PMO-M	0.001	two-tailed t test
	A	PMO v.s PMO-M	0.015	two-tailed t test
	D	PMO v.s PMO-M	0.020	two-tailed t test
Fig.3F	TA	PMO v.s PMO-M	0.1	two-tailed t test
	Q	PMO v.s PMO-M	0.002	two-tailed t test
	G	PMO v.s PMO-M	0.002	two-tailed t test
	T	PMO v.s PMO-M	0.1	two-tailed t test
	A	PMO v.s PMO-M	0.013	two-tailed t test
	D	PMO v.s PMO-M	0.1	two-tailed t test
Fig.3H	TA	PMO v.s PMO-M	0.218	two-tailed t test
	Q	PMO v.s PMO-M	0.044	two-tailed t test
	G	PMO v.s PMO-M	0.024	two-tailed t test
	T	PMO v.s PMO-M	0.115	two-tailed t test
	A	PMO v.s PMO-M	0.7	two-tailed t test
	D	PMO v.s PMO-M	0.506	two-tailed t test
Fig.4C	TA	PMO v.s PMO-M	0.00065	two-tailed t test
	Q	PMO v.s PMO-M	0.00091	two-tailed t test
	G	PMO v.s PMO-M	2.85E-05	two-tailed t test
	T	PMO v.s PMO-M	0.000363	two-tailed t test
	A	PMO v.s PMO-M	0.03	two-tailed t test
	D	PMO v.s PMO-M	0.000276	two-tailed t test
Fig.4E	TA	PMO v.s PMO-M	0.019	two-tailed t test
	Q	PMO v.s PMO-M	0.002	two-tailed t test
	G	PMO v.s PMO-M	0.006	two-tailed t test
	T	PMO v.s PMO-M	0.001	two-tailed t test
	A	PMO v.s PMO-M	0.004	two-tailed t test
	D	PMO v.s PMO-M	0.030	two-tailed t test
Fig.4F	6 Weeks	mdx v.s PMO	0.469	One way-ANOVA post hoc Student-Newman-Keuls test
		mdx v.s PMO-M	0.021	One way-ANOVA post hoc Student-Newman-Keuls test
		PMO v.s PMO-M	0.032	One way-ANOVA post hoc Student-Newman-Keuls test

	10 Weeks	mdx v.s PMO	0.412	One way-ANOVA post hoc Student-Newman-Keuls test
		mdx v.s PMO-M	0.004	One way-ANOVA post hoc Student-Newman-Keuls test
		PMO v.s PMO-M	0.006	One way-ANOVA post hoc Student-Newman-Keuls test
	14 Weeks	mdx v.s PMO	0.22	One way-ANOVA post hoc Student-Newman-Keuls test
		mdx v.s PMO-M	0.000959	One way-ANOVA post hoc Student-Newman-Keuls test
		PMO v.s PMO-M	0.000912	One way-ANOVA post hoc Student-Newman-Keuls test
	16 Weeks	mdx v.s PMO	0.749	One way-ANOVA post hoc Student-Newman-Keuls test
		mdx v.s PMO-M	0.002	One way-ANOVA post hoc Student-Newman-Keuls test
		PMO v.s PMO-M	0.002	One way-ANOVA post hoc Student-Newman-Keuls test
Fig.4G		mdx v.s PMO	0.688	One way-ANOVA post hoc Student-Newman-Keuls test
		mdx v.s PMO-M	0.025	One way-ANOVA post hoc Student-Newman-Keuls test

		PMO v.s PMO-M	0.021	One way-ANOVA post hoc Student-Newman-Keuls test
Fig.5A		mdx v.s PMO	0.754	One way-ANOVA post hoc Student-Newman-Keuls test
		mdx v.s PMO-M	0.026	One way-ANOVA post hoc Student-Newman-Keuls test
		PMO v.s PMO-M	0.019	One way-ANOVA post hoc Student-Newman-Keuls test
Fig.5D	Quadriceps	mdx v.s PMO	0.461	One way-ANOVA post hoc Student-Newman-Keuls test
		mdx v.s PMO-M	4.44E-05	One way-ANOVA post hoc Student-Newman-Keuls test
		PMO v.s PMO-M	1.92E-05	One way-ANOVA post hoc Student-Newman-Keuls test
	Triceps	mdx v.s PMO	0.884	One way-ANOVA post hoc Student-Newman-Keuls test
		mdx v.s PMO-M	0.000462	One way-ANOVA post hoc Student-Newman-Keuls test
		PMO v.s PMO-M	0.000131	One way-ANOVA post hoc Student-Newman-Keuls test
Fig.5E	<500	PMO v.s PMO-M	0.13	two-tailed t test
	500-1000	PMO v.s PMO-M	0.042	two-tailed t test
	1000-1500	PMO v.s PMO-M	0.363	two-tailed t test
	1500-2000	PMO v.s PMO-M	0.383	two-tailed t test

	2000-2500	PMO v.s PMO-M	0.881	two-tailed t test
	2500-3000	PMO v.s PMO-M	0.15	two-tailed t test
	3000-3500	PMO v.s PMO-M	0.224	two-tailed t test
	3500-4000	PMO v.s PMO-M	0.543	two-tailed t test
	4000-4500	PMO v.s PMO-M	0.434	two-tailed t test
	>4500	PMO v.s PMO-M	0.870	two-tailed t test
Fig.6A	AST	mdx v.s PMO	0.859	One way-ANOVA post hoc Student-Newman-Keuls test
		mdx v.s PMO-M	0.019	One way-ANOVA post hoc Student-Newman-Keuls test
		PMO v.s PMO-M	0.016	One way-ANOVA post hoc Student-Newman-Keuls test
	ALT	mdx v.s PMO	0.84	One way-ANOVA post hoc Student-Newman-Keuls test
		mdx v.s PMO-M	0.005	One way-ANOVA post hoc Student-Newman-Keuls test
		PMO v.s PMO-M	0.014	One way-ANOVA post hoc Student-Newman-Keuls test
	GGT	mdx v.s PMO	0.753	One way-ANOVA post hoc Student-Newman-Keuls test
		mdx v.s PMO-M	0.051	One way-ANOVA post hoc Student-Newman-Keuls test
		PMO v.s PMO-M	0.288	One way-ANOVA post hoc Student-Newman-Keuls test
Fig.6E	CD68-Quadriceps	mdx v.s PMO	0.012	One way-ANOVA post hoc

				Student-Newman-Keuls test
		mdx v.s PMO-M	0.00083	One way-ANOVA post hoc Student-Newman-Keuls test
		PMO v.s PMO-M	0.003	One way-ANOVA post hoc Student-Newman-Keuls test
	CD68-Diaphgram	mdx v.s PMO	0.370	One way-ANOVA post hoc Student-Newman-Keuls test
		mdx v.s PMO-M	0.003	One way-ANOVA post hoc Student-Newman-Keuls test
		PMO v.s PMO-M	0.005	One way-ANOVA post hoc Student-Newman-Keuls test
	CD11b-Quadriceps	mdx v.s PMO	0.011	One way-ANOVA post hoc Student-Newman-Keuls test
		mdx v.s PMO-M	0.0009446	One way-ANOVA post hoc Student-Newman-Keuls test
		PMO v.s PMO-M	0.005	One way-ANOVA post hoc Student-Newman-Keuls test
	CD11b-Diaphgram	mdx v.s PMO	0.208	One way-ANOVA post hoc Student-Newman-Keuls test
		mdx v.s PMO-M	0.009	One way-ANOVA post hoc Student-Newman-Keuls test
		PMO v.s PMO-M	0.027	One way-ANOVA post hoc

				Student-Newman-Keuls test
	CD3-Quadriceps	mdx v.s PMO	0.804	One way-ANOVA post hoc Student-Newman-Keuls test
		mdx v.s PMO-M	0.021	One way-ANOVA post hoc Student-Newman-Keuls test
		PMO v.s PMO-M	0.013	One way-ANOVA post hoc Student-Newman-Keuls test
	CD3-Diaphgram	mdx v.s PMO	0.754	One way-ANOVA post hoc Student-Newman-Keuls test
		mdx v.s PMO-M	0.049	One way-ANOVA post hoc Student-Newman-Keuls test
		PMO v.s PMO-M	0.035	One way-ANOVA post hoc Student-Newman-Keuls test
Fig.EV1	TA	PMO-M vs PMO	0.005	t test
	G	PMO-M vs PMO	0.008	t test
Fig.EV2B		PMO-M vs PMO	0.0009125	One way-ANOVA post hoc Student-Newman-Keuls test
		PMO-M vs. Seperated	0.130	One way-ANOVA post hoc Student-Newman-Keuls test
		Seperated vs. PMO	0.0012	One way-ANOVA post hoc Student-Newman-Keuls test
Fig.EV2D		PMO-M vs PMO	0.004	One way-ANOVA post hoc Student-Newman-Keuls test

		PMO-M vs. Seperated	0.649	One way-ANOVA post hoc Student-Newman-Keuls test
		Seperated vs. PMO	0.003	One way-ANOVA post hoc Student-Newman-Keuls test
Fig. EV3B	Soleus	PMO-M vs PMO	0.005	two-tailed t test
	EDL	PMO-M vs PMO	0.014	two-tailed t test
Fig. EV4B	Soleus	PMO-M vs PMO	0.0012	two-tailed t test
	EDL	PMO-M vs PMO	0.003	two-tailed t test
Fig.EV5	0 week	PMO-M vs PMO-M(+MOTS-c)	0.529	two-tailed t test
	1 week	PMO-M vs PMO-M(+MOTS-c)	0.637	two-tailed t test
	2 week	PMO-M vs PMO-M(+MOTS-c)	0.18	two-tailed t test
	4 week	PMO-M vs PMO-M(+MOTS-c)	0.017	two-tailed t test
	Body weight gain	PMO-M vs PMO-M(+MOTS-c)	0.031	two-tailed t test
Appendix S1C	TA	PMO-M vs PMO-GF	0.09	two-tailed t test
	Q	PMO-M vs PMO-GF	0.003	two-tailed t test
	G	PMO-M vs PMO-GF	0.003	two-tailed t test
	T	PMO-M vs PMO-GF	0.106	two-tailed t test
	A	PMO-M vs PMO-GF	0.154	two-tailed t test
	D	PMO-M vs PMO-GF	0.387	two-tailed t test
Appendix S3C	TA	PMO-M vs PMO-G	0.048	two-tailed t test
	Q	PMO-M vs PMO-G	0.011	two-tailed t test
	G	PMO-M vs PMO-G	0.005	two-tailed t test
	T	PMO-M vs PMO-G	0.122	two-tailed t test
	A	PMO-M vs PMO-G	0.000465	two-tailed t test
	D	PMO-M vs PMO-G	0.061	two-tailed t test
Appendix S4D	Quadriceps	mdx vs MOTS-c	0.001	two-tailed t test
	Diaphragm	mdx vs MOTS-c	0.008	two-tailed t test
Appendix S4E		mdx vs. MOTS-c	0.027	One way-ANOVA post hoc Student-Newman-Keuls test
		mdx vs. C57	0.304	One way-ANOVA post hoc Student-Newman-Keuls

				test
		C57 vs. MOTS-c	0.062	One way-ANOVA post hoc Student-Newman-Keuls test
