# Supplementary material for: MOTS‐c promotes phosphorodiamidate morpholino oligomer uptake and efficacy in dystrophic mice
Source: EMBO Mol Med. 2020 Dec 18;13(2):e12993. doi: 10.15252/emmm.202012993 (PMC7863382; doi:10.15252/emmm.202012993)
Supplement: Supplementary file 2 — Expanded View Figures PDF [file EMMM-13-e12993-s002.pdf]

Expanded View Figures

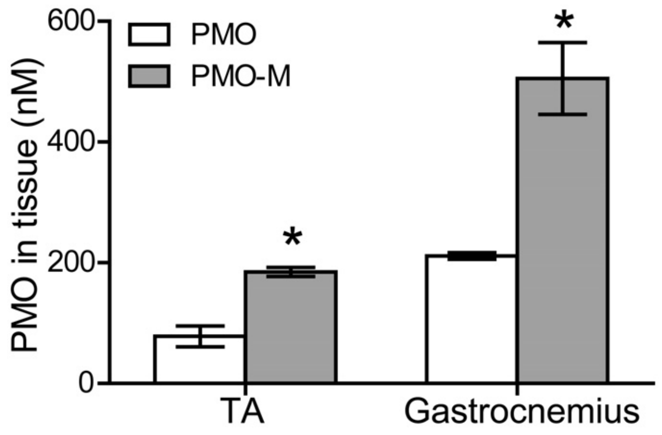

Figure EV1. Measurement of PMO in muscle tissues of *mdx* mice.
FITC-labeled PMO (50 mg/kg) mixed with MOTS-c (500 μg) (PMO-M) (n = 3) or FITC-labeled PMO in saline (PMO) (n = 3) was intravenously injected into *mdx* mice for once, and tissues were harvested 48 h later (*$P < 0.05$, two-tailed t-test). TA—tibialis anterior.
Data information: Data were presented as mean ± sem. Exact P values are specified in Appendix Table S1.

 © 2021 The Authors

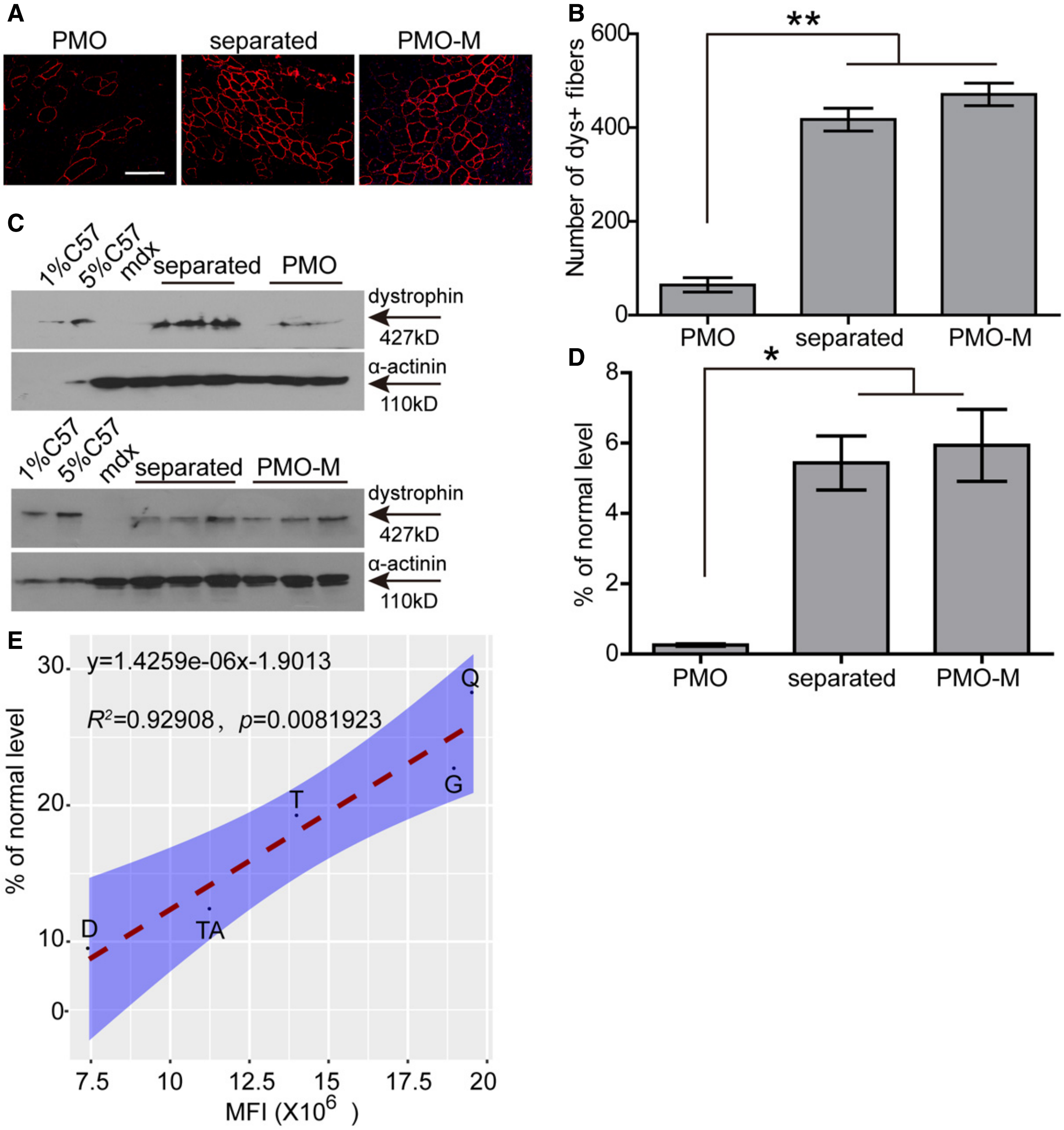

Figure EV2. Evaluation of the direct effect of MOTS-c on PMO activity in *mdx* mice and correlation of efficacy/quantity of PMO in dystrophic muscles.

PMO (0.5 μg) in saline was administered into TA muscles of adult *mdx* mice, and MOTS-c (500 μg) was simultaneously injected into the same mice intravenously, and muscles were harvested 2 weeks after injection.

A, B Immunohistochemistry (A) and quantitative analysis (B) of dystrophin-positive fibers in TA muscles from treated *mdx* mice (scale bar: 100 μm) ($n = 3$; $^{**}P < 0.001$, one-way ANOVA post hoc Student–Newman–Keuls test). PMO-M means PMO mixed with MOTS-c, and PMO refers to PMO alone (the same is for the rest unless otherwise specified). Separated refers to intramuscular injection of PMO with simultaneous systemic injection of MOTS-c.

C, D Western blot (C) and quantitative analysis (D) of dystrophin expression in TA muscles from treated *mdx* mice ($n = 3$; $^{*}P < 0.05$, one-way ANOVA post hoc Student–Newman–Keuls test). 0.5 μg and 2.5 μg total protein from *C57BL/6* and 50 μg of muscle samples from untreated and treated *mdx* mice were loaded. α-actinin was used as the loading control.

E Analysis of the correlation between quantity and efficacy of PMO in dystrophic muscles following systemic injection. Adult *mdx* mice were treated with PMO-M intravenously at the PMO dose of 50 mg/kg/week for 3 weeks, and muscles were harvested two weeks after last injection to examine dystrophin expression. Fluorescence intensity was measured 48 h after single intravenous injection of FITC-labeled PMO (50mg/kg) into adult *mdx* mice. TA—tibialis anterior, Q—quadriceps, G—gastrocnemius, T—triceps, D—diaphragm.

Data information: Data were presented as mean ± sem. Exact *P* values are specified in Appendix Table S1.

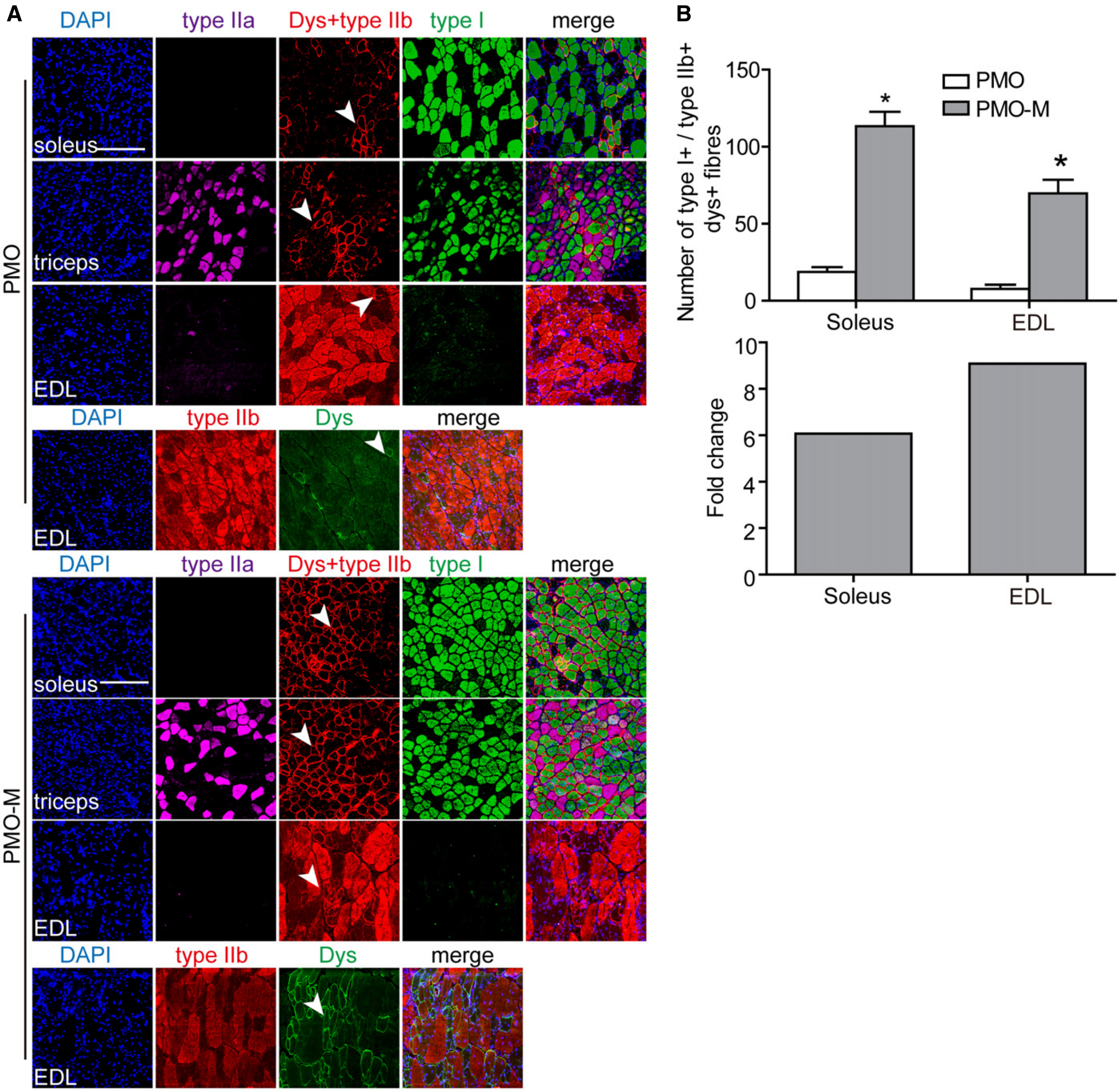

Figure EV3. Examination of muscle fiber type-specific dystrophin expression in treated *mdx* mice.

Adult *mdx* mice were intravenously injected with PMO at the dose of 12.5 mg/kg/week for 3 weeks mixed with MOTS-c (500 μg), and tissues were harvested and examined two weeks after last injection.

A Immunohistochemistry for dystrophin expression in slow-twitch (soleus), fast-twitch (EDL), and mixed type (triceps) of muscle fibers of treated *mdx* mice (scale bar: 100 μm). Type I MHC was used to identify slow-twitch muscle fibers, and type IIa and IIb MHC were used to characterize fast-twitch muscle fibers. The arrowheads point to dystrophin-positive fibers.

B Quantitative analysis of type I MHC- and dystrophin-positive or type IIb MHC- and dystrophin-positive fibers in treated *mdx* mice ($n = 3$; $^*P < 0.05$, two-tailed *t*-test). Type I^+ or type IIb^+ means type I MHC-positive or type IIb MHC-positive fibers, respectively. Dys^+ represents dystrophin-positive fibers. Fold change refers to PMO-M relative to PMO alone.

Data information: Data were presented as mean ± sem. Exact *P* values are specified in Appendix Table S1.

 © 2021 The Authors

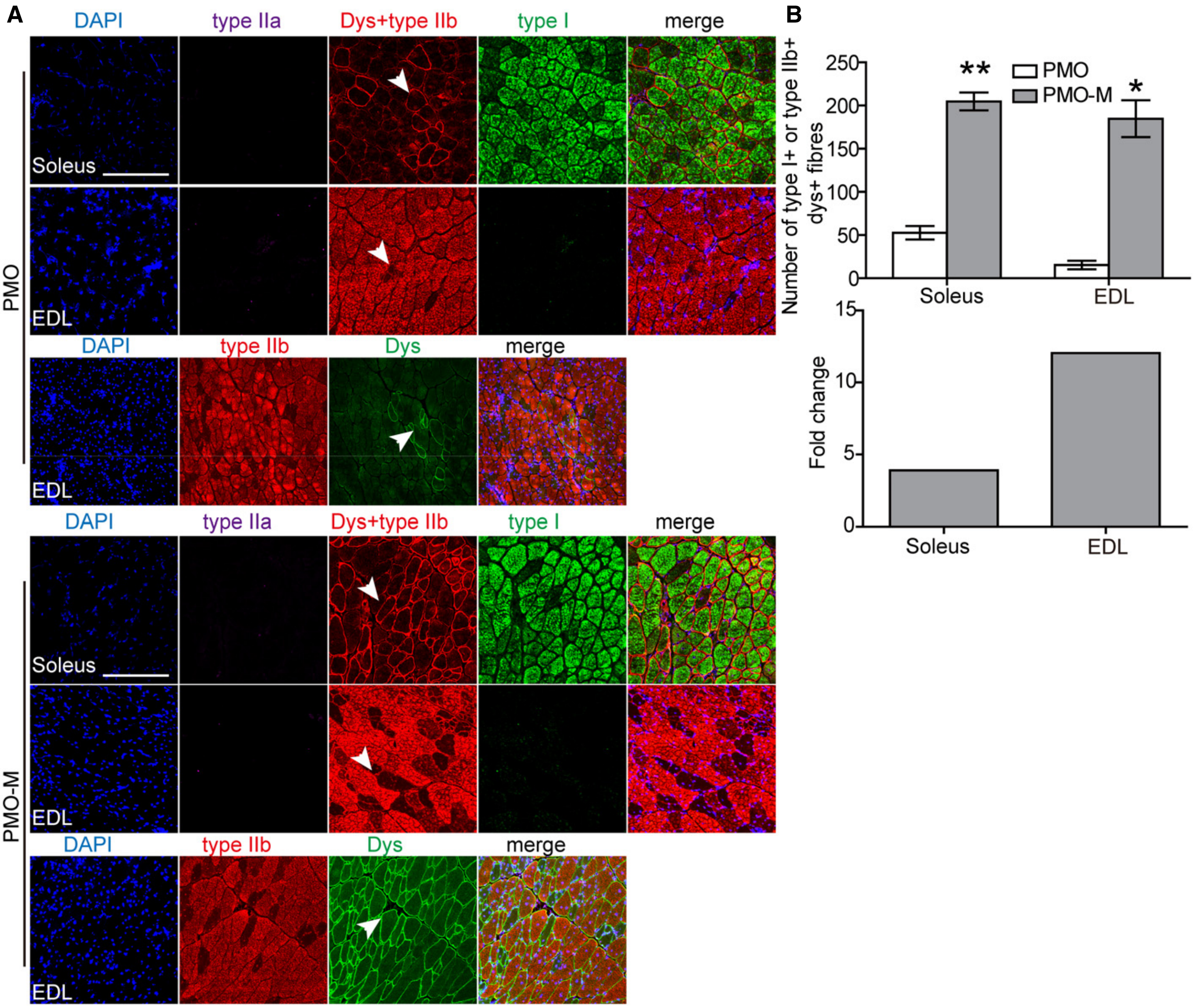

Figure EV4. Effect of PMO-M on dystrophin expression in different muscle fiber types of treated *mdx* mice.

PMO-M was administered intravenously into adult *mdx* mice at the PMO dose of 12.5 mg/kg/week for 3 weeks followed by 12.5 mg/kg/month for 3 months, and tissues were harvested two weeks after last injection.

A Immunohistochemistry for dystrophin expression in slow-twitch (soleus) and fast-twitch (EDL) muscle fibers of treated *mdx* mice (scale bar: 100 μm). Type I MHC was used to identify slow-twitch muscle fibers, and type IIa and IIb MHC were used to characterize fast-twitch muscle fibers. The arrowheads point to dystrophin-positive fibers.

B Quantitative analysis of type I MHC- and dystrophin-positive or type IIb MHC- and dystrophin-positive fibers in *mdx* mice treated with PMO-M ($n = 4$) or PMO ($n = 3$) (*$P < 0.05$, **$P < 0.001$, two-tailed t-test). Type I^+ or type IIb^+ means type I MHC-positive or type IIb MHC-positive fibers, respectively. Dys^+ represents dystrophin-positive fibers. Fold change refers to PMO-M relative to PMO alone.

Data information: Data were presented as mean ± sem. Exact P values are specified in Appendix Table S1.

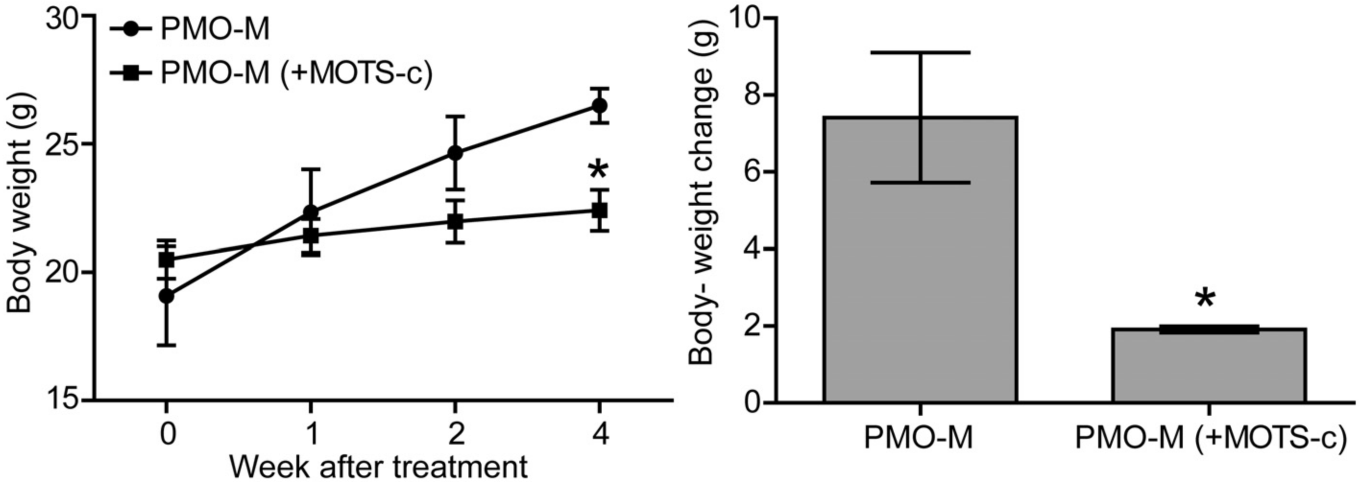

Figure EV5. Measurement of body-weight changes of treated *mdx* mice.

Adult *mdx* mice were treated with PMO-M or PMO-M with additional MOTS-c supply (500 μg) (PMO-M (+MOTS-c)) at the PMO dose of 50 mg/kg/week for 3 weeks intravenously (n = 3; *$P < 0.05$, two-tailed t-test).

Data information: Data were presented as mean ± sem. Exact P values are specified in Appendix Table S1.

 © 2021 The Authors
